# It’s Worth What You Can Sell It for: A Survey of Employment and Compensation Models for Clinical Ethicists

**DOI:** 10.1007/s10730-023-09509-y

**Published:** 2023-08-05

**Authors:** Jason Adam Wasserman, Abram Brummett, Mark Christopher Navin

**Affiliations:** 1 Department of Foundational Medical Studies, Oakland University William Beaumont School of Medicine, Rochester, MI USA; 2 Department of Pediatrics, Oakland University William Beaumont School of Medicine, Royal Oak, MI USA; 3 Clinical Ethics, Corewell Health William Beaumont University Hospital, Royal Oak, MI USA; 4 Department of Philosophy, Oakland University, Rochester, MI USA

**Keywords:** Clinical ethics, Employment, Compensation, Professionalization

## Abstract

This article reports results of a survey about employment and compensation models for clinical ethics consultants working in the United States and discusses the relevance of these results for the professionalization of clinical ethics. This project uses self-reported data from healthcare ethics consultants to estimate compensation across different employment models. The average full-time annualized salary of respondents with a clinical doctorate is $188,310.08 (SD=$88,556.67), $146,134.85 (SD=$55,485.63) for those with a non-clinical doctorate, and $113,625.00 (SD=$35,872.96) for those with a masters as their highest degree. Pay differences across degree level and type were statistically significant (F = 3.43; p < .05). In a multivariate model, there is an average increase of $2,707.84 for every additional year of experience, controlling for having a clinical doctorate (ß=0.454; p < .01). Our results also show high variability in the backgrounds and experiences of healthcare ethics consultants and a wide variety of employment models. The significant variation in employment and compensation models is likely to pose a challenge for the professionalization of healthcare ethics consultation.

## Introduction

The 1976 ruling of the New Jersey Supreme Court in the case of Karen Ann Quinlan directed hospitals to establish ethics committees to resolve ethically complicated cases before they reached the courts (In re Quinlan, 1976). The establishment of clinical ethics services was further bolstered by the passage of the Patient Self-Determination Act in 1991 and the 1992 stipulation by the Joint Commission on Accreditation of Healthcare Organizations that member institutions must have a “mechanism” for addressing moral uncertainty or conflict that arises during patient care. While the Joint Commission does not require ethics consultation services per se, its current language states that, “The organization develops and implements a process that allows staff, patients, and families to address ethical issues or issues prone to conflict” (Joint Commission, 2023). Especially among larger institutions, this duty is typically discharged by establishing an ethics consultation service (Fox, Danis, Tarzian, and Duke, 2021a). However, the staffing, roles, and processes of these services vary widely.

Clinical ethicists have varied backgrounds and engage in diverse activities. This pluralism has often been regarded as a strength of the field. Clinical ethics practice has been traditionally shaped by physicians, nurses, social workers, and chaplains; but theologians, attorneys, and philosophers have also been involved (Fox, Myers, and Pearlman, 2007). The work of healthcare ethics consultation often includes education, policy formation and review, and case consultation. There are different models for case consultation, including reviews conducted by small teams of individuals, full ethics committees, and single consultants (Fox et al., 2007). After the Joint Commission required that hospitals have a means for addressing ethical conflict, many textbooks, handbooks, and guidelines were produced by organizations such as the American Society for Bioethics and Humanities, the Hastings Center, and others. Their efforts illustrate that the field of healthcare ethics consultation developed sets of best practices, though debates continue about many topics (see Brummett and Watson, 2022). However, adoption of these best practices has not been as widespread as expected, and Tarzian et al. (2022, 11) have suggested using a “rigorous consensus process to develop a new EC standards document that provides clear, unambiguous guidance.” Perhaps the strongest indicator of this trend toward professionalization is the Healthcare Ethics Consultant-Certified (HEC-C) program (Horner, Childress, Fantus, and Malek, 2020).

The professionalization of healthcare ethics consultation raises questions about its value for the institutions it serves. The primary value of healthcare ethics consultation, of course, is to promote more ethical and just patient care and to negotiate values conflicts to preserve the integrity of patients, families, and staff. However, clinical ethics practice also has *financial* value for institutions and this value should not be disregarded, particularly in discourses surrounding the professionalization of clinical ethics.

Some studies have identified substantial cost savings associated with ethics case consultation. For example, patients who receive clinical ethics consultation spend fewer days in the hospital, but have no difference in overall mortality compared to patients who did not receive a consultation (Schneiderman et al., 2003; Schneiderman, 2000, 2006; Dowdy, 1998). Fewer days spent in the hospital usually results in lower institutional costs (Repenshek, 2017). One study estimated that a hospital with 485 total beds (40 of which were in the ICU) would save an average of $157,380 per year by having an ethics service (Gilmer, 2005, 969). However, many of the studies that identify the direct cost savings associated with healthcare ethics consultation are nearly two decades old. Moreover, predicting cost savings associated with clinical ethics consultation is riddled with potential confounding factors, such as selectivity bias for kinds of cases on which ethics services get consulted. Thus, this evidence may not be as persuasive now as it once was and it was always owed at least some degree of skepticism.

In light of the many questions about measuring the value of ethics by its associated cost savings, another way to identify the value of healthcare ethics consultation is to reveal what healthcare institutions are willing to pay for it. This study aims to show how much money those institutions are willing to invest in healthcare ethics consultants. In doing so, we simultaneously address two important issues about the financial value of healthcare ethics consultation in the context of the professionalization of that practice:

1. Estimating compensation levels across different employment models of healthcare ethics consultants, accounting for factors such as education, years of experience, and primary discipline;
2. Estimating the value of the multiple functions of healthcare ethics consults, rather than only those related to case consultation (the sole function evaluated by cost savings data described above).

To accomplish the above, we first describe our data collection process, including the measures we used to specify the different functions of healthcare ethics consultants and the varied employment models within which they work. We then report the different employment arrangements and individual characteristics of respondents to identify salary point estimates. We also report other kinds of institutional support offered to healthcare ethics consultants, as well as consultants’ perceptions of their compensation. We conclude by discussing the implications of these data for the professionalization of the field.

## Methods

### Survey and Sample

In Fall of 2020, an anonymous online survey was developed within the Qualtrics platform and tested among a small subset of healthcare ethics consultants within our state network who provided feedback. It was then was distributed to the listserv for the Clinical Ethics Consultation Affinity Group (CECAG), which is affiliated with the American Society for Bioethics and Humanities. Founded in 2008, CECAG supports a private, moderated listserv of more than 1000 clinical ethicists and others interested in the field; it is a place to share information and deliberate about the profession (Kipnis, 2009; T. V. Cunningham, personal communication, March 1st, 2023). The survey was initially posted to the listserv on October 19, 2020 and closed on November 17, 2020. Recipients were encouraged to share the survey with other clinical ethicists, even outside of the CECAG membership. The study was approved by the Oakland University Institutional Review Board.

The initial item screened for was whether the respondent provided services in healthcare ethics consultation, including case consultation, policy review, or education. Individuals who did not report active participation in healthcare ethics consultation were routed directly to survey termination. Individuals who did not complete the survey, defined *a priori* as having greater than 10% of items unanswered, were excluded from the analytic data set (though in practical terms, all excluded surveys were nearly totally incomplete sans a few initial questions, and therefore their exclusion did not have any appreciable effect on validity of the data). This yielded 133 completed responses. (Full survey text is available by request of the corresponding author.)

## Measures

Many survey items allowed respondents to select multiple options, which were then collapsed into analyzable composite variables. The measures utilized in the analyses below are as follows:

### Demographics

The survey solicited basic respondent characteristics such as gender identity with response choices of “Male,” “Female,” and “Additional Category” which allowed respondents to write in the description they felt most appropriate.^1^ Age and consultation experience were both measured in years.

### Highest Degree

Based on respondents’ indications of all earned degrees, a “Highest Degree” variable was created from respondent’s degree selections, prioritizing those with MD or DO as “Clinical Doctorate,” those with PhD, DBe and the like, but no medical degree, as “Non-clinical Doctorate,”^2^ and those whose highest degree was a “Masters” or “Bachelors.” We also solicited a binary (yes/no) response as to whether any degree held by the respondent was focused specifically on bioethics or clinical bioethics. Along with a question about non-degree related clinical ethics training, this allowed us to estimate the amount of formal clinical ethics education.

### HEC-C (Planned or held)

Those who selected the HEC-C as one of their credentials were combined in some analyses with those who reported planning to pursue that credential within the next year to create a composite “have or plan to get HEC-C” variable.

### Primary Professional Role

One of the challenges of studying clinical ethicists is that their healthcare ethics consultation work is often an adjunctive endeavor to other professional roles. To capture the diversity of professional roles in an analyzable way, respondents were able to select multiple roles, as well as writing in their role in a residual response choice. These responses were then collapsed into “Clinical Ethics” (for those selecting clinical ethics (employed), clinical ethics fellow, or writing in an answer that indicated clinical ethics work as a professional role, such as serving on an ethics committee); “Clinical Care” (for those selecting physician, physician assistant, nurse practitioner, nurse, or writing in another clinical care role, e.g. psychologist or physical therapist); “Clinical Support” (for those selecting chaplaincy or social work); “Administrative” (for those selecting administrator or writing in a response that indicated administrative work such as legal counsel or mission leader); and “Academic Faculty Appointments” (for those selecting academic faculty as a role). In order to present a complete picture of the kinds of professional roles occupied by clinical ethicists in our sample, we present the prevalence of responses in each category, as well as joint frequencies of the most common types.

### Employment Model

Respondents self-selected their clinical ethics employment model by choosing from one of the following categories: “Full Time”, “Part Time”, “Partial Buyout” (for those whose ethics consultation work is formally partitioned from their regular job duties in another field), “retainer” (for those who receive a flat prepaid fee), “Hourly” (for those compensated for ethics consultation on an hourly basis), and “Other” which allowed respondents to describe an alternate employment model (this was particularly used to capture those who felt healthcare ethics consultation was a form of service inherent in their job duties). Each of these were followed with questions about different rates, as appropriate, and then ultimately annualized as full-time equivalent values in many of the analyses below, based on a standard 2080-hour work year.

### Consultation Service Characteristics

This is a set of variables including “Average Monthly Consult Volume” (measured as the number of consults performed each month “in a normal year (i.e. COVID notwithstanding”); “Evening or Weekend Call” (measured as a binary indicating whether or not the respondent is ever on call during evenings or weekends); and “Consult Service Workload Allocation” (measured as the percent allocation of clinical ethics services across case consultation, education, and policy review, with a residual category allowing respondents to write in other kinds of clinical ethics work, e.g. administrative functions).

### Other Variables

“Other Clinical Ethics Training,” “Geographical Setting,” and “Healthcare Setting” included response options as seen in Table 1 below, and were not further modified. “Other Forms of Support” for healthcare ethics consultation work allowed respondents to select whether employers provided development or travel reimbursement; liability insurance; office space; a work computer; a work phone or tablet; compensation for the HEC-C exam fee; or to write in other benefits, compensation, or forms of support. Respondents also were asked about their general satisfaction with their compensation using a 7-point Likert scale ranging from “Extremely Satisfied” to “Extremely Dissatisfied”, with a neutral middle option.

### Analyses

Most data analyses involved descriptive statistics aimed at providing point estimates and ranges for compensation, both overall and within different employment arrangements. Because of the different employment models in healthcare ethics consultation, the diverse educational and professional backgrounds of healthcare ethics consultants, and the preponderance of healthcare ethics consultants to work in multiple capacities, many of the descriptive analyses must be read as reflecting prevalences within categories, but not as zero-sum estimates of the roles and functions within healthcare ethics consultation as a whole. For some variables, the most prevalent open-ended responses to residual response choices are reported. Analysis was performed in SPSS v.28.0.0.0.

Bivariate and multivariate analyses were employed to highlight the influence of some factors (e.g., clinical versus non-clinical highest degree) on pay rate and having or planning to pursue the HEC-C certification. Bivariate tests involved chi-square analysis to examine differences in observed versus expected joint frequencies (e.g., difference in proportions of compensated versus uncompensated clinical ethicists who have/plan to get the HEC-C). Analysis of variance (ANOVA) was used to test differences in means between groups (e.g., differences in compensation by highest degree). A multivariate model predicting pay was analyzed using ordinary least squares regression. Responses to open-ended questions were coded systematically using a basic narrative coding technique that engaged in line-by-line coding and subsequent categorization of like codes to identify patterns (Lofland and Lofland, 1995).

## Results

### Demographics

In total, 147 respondents initiated the survey. Of those, 2 respondents did not agree to participate after reading the consent form, 1 indicated that they did not actively participate in healthcare ethics consultation, and 11 failed to sufficiently complete the survey (> 90% of questions answered). This yielded an analytic sample of 133 cases (tables indicate the sample sizes used in each analysis after accounting for missing data points).

Key descriptive results are summarized in Table 1. Importantly, though perhaps unsurprisingly, there is a statistically significant difference between observed and expected joint frequencies of having a bioethics focused degree across the highest degree categories. This was driven by an overrepresentation of those with non-clinical doctorates who had a bioethics focused degree (𝝌^2^ = 13.24; p < .01). Further, 50.9% of respondents either have the HEC-C credential or plan to take the credentialing exam in the next year.

**Table 1:** Sample Characteristics (n = 133, unless otherwise specified)

**Sex/Gender Identity**		
	Male	51 (37.6%)
	Female	75 (56.4%)
	Non-Binary	1 (0.75%)
	Did Not Answer	6 (4.5%)
**Mean Age (SD) (Range)**		52.45 (12.0) (31–81)
**Highest Degree (n = 129)** **[Number and % within category holding any kind of bioethics-focused degree]**^**1**^		
	MD/DO	30 (23.3%) [8 (26.7%)]
	PhD	75 (58.1%) [48 (64.0%)]
	Masters	23 (17.8%) [13 (56.5%)]
	Bachelors	1 (0.8%) [0 (0.00%)]
		χ2 = 13.24; p < .01
**HEC-C**		
	Currently held	38 (28.6%)
	Plan to get	29 (21.8%)
	Combined (have or intend to get)	67 (50.4%)
**Percent with Other Clinical Ethics Training** (n = 70/129; 54.7%)		
	One-year Fellowship	25 (35.7%)
	Two-year Fellowship	16 (22.9%)
	Certificate Program	11 (15.7%)
	Short-term Intensive Course	11 (15.71%)

^1^Indicates percent within the category of highest degree that hold any kind of bioethics focused degree, not the percent of highest degrees that are bioethics-focused

### Practice Setting

Table 2 shows results highlighting the vast majority of respondents working in large cities or suburbs. With regard to health care setting, the vast majority of the sample worked in teaching or community hospitals and for non-profit institutions.

**Table 2:** Practice Setting (n = 127)

**Geographical Setting**		
	Large City	79 (62.2%)
	Suburb	25 (19.7%)
	Small City	21 (16.5%)
	Rural	2 (1.6%)
**Healthcare Setting**^1^		
	Teaching Hospital	97 (76.4%)
	Community Hospital	46 (36.2%)
	Government Hospital	7 (5.5%)
	Ambulatory Site	18 (14.2%)
	Long-term Care Facility	11 (8.7%)
	Psychiatric Hospital	10 (7.9%)
	Non-Profit	85 (66.9%)
	For-Profit	2 (1.6%)

^1^Percentages do not total to 100% because respondents can work at multiple locations and some institutions represent multiple categories

### Professional Roles, Experience, and Service Characteristics

Table 3 highlights the diverse professional roles occupied by clinical ethicists. Indeed, those serving in primarily clinical ethics roles are less than half of the total sample, with a substantial portion having a primarily clinical role. Importantly, substantial portions of those with administrative roles also provide clinical care and direct clinical ethics consultation. A comparatively small percentage of the sample come from clinical support such as social work or pastoral care. Additionally, case consultation makes up nearly half of the reported clinical ethics workload and the vast majority take call on evenings or weekends.

**Table 3:** Professional Roles, Experience, and Service Characteristics

**Professional Roles Occupied (n = 129)**	
Clinical Ethics	66 (49.6%)
Clinical Care	43 (32.3%)
Clinical Support	7 (5.3%)
Administrative (total)^1^	26 (19.6%)
and Clinical Care	7 (5.3%)
and Clinical Ethics	19 (14.3%)
and Clinical Support	1 (0.8%)
Academic Faculty Appointments (total)^1^	71 (53.4%)
with no other role	15 (11.3%)
and Clinical Care	25 (18.8%)
and Clinical Ethics	36 (27.1%)
and Administrative	10 (7.5%)
and Clinical Support	1 (0.8%)
**Mean Years of Consultation Experience (SD) (Range)**	16.09 (10.17) (1–36)
**Consult Service Characteristics**	
Average Monthly Volume (SD) (Range) (n = 125)	8.54 (7.22) (0–35)
Percent taking Evening/Weekend Call (n = 127)	95 (71.4%)
Days per Month Evening/Weekend Call (SD) (Range)	15.56 (11.92) (1–31)
Percent Workload Allocation (SD) (n = 129)	
Consultation	44.7% (26.20)
Education	25.9% (18.16)
Policy Review	16.1% (17.01)

^1^Joint roles are greater than total because of a small number of individuals occupying more than two professional roles and another small number listing no joint professional roles

### Compensation Models

#### Annualized Compensation

An average annualized salary (at 2,080 work hours per year) was $153,192.99 (SD=$79,234.98). However, with two outliers earning greater than $400,000 per year removed to correct for intolerable skewness in this variable, the distribution normalizes around $143,044.47 (SD=$57,688.43). Because of the high degree of variation in this overall estimate, we partition salary estimates by degree type and employment model in Table 4 below.

**Table 4:** Salary Point Estimates

	**Mean (SD)**
**Annualized Full Time Equivalent (FTE)1 (n = 60)**	$153,192.99 (79,234.98)
Outliers removed^2^ (n = 58)	$143,044.47 (57,688.43)
**Annualized Compensation by Degree Type**^3^	
Clinical Doctorate (n = 5)	$188,310.08 (88,556.67)
Non-Clinical Doctorate (n = 41)	$146,134.85 (55,485.63)
Masters (n = 12)	$113,625.00 (35,872.96)
	F = 3.43; p < .05
**Compensation by Employment Model**^4^	
Full Time Clinical Ethicist (n = 46)	$135,702.35 (51,340.01)
Clinical Doctorate (n = 1)	$85,000 (NA)
Non-Clinical Doctorate (n = 36)	$141,494.67 (53,466.09)
Masters (n = 9)	$118,166.67 (38,750.81)
Partial buyout (n = 11)^5^	$55,663.64 (39,114.12)
Average Percent of FTE Bought Out (n = 12)	30.0% (22.6%)
Retainer (n = 6)	$3,260/mo (4,934.13)
Hourly (n = 7)	$170.83/hr (65.99)

^1^ Calculated as the salary of those working full time in clinical ethics or the full-time salaries of those working on partial buy-out

^2^ Two reported salaries from the buy-out category were substantially greater than the remainder of the group and increased the skewness statistic to an intolerable degree. These were removed where indicated

^3^ Two outliers removed for this analysis

^4^ Annualized unless otherwise noted

^5^ The “Highest Degree” subgroups are not displayed in the table because of the small subgroup size

#### Compensation by Degree Type

As shown in Table 4, average annualized salary varies significantly across degree type (F = 3.43; p < .05), with the difference being primarily attributable to the distinction between those with a highest degree as clinical doctorate and those reporting a masters as their highest degree. In a multivariate model, there is an average increase of $2,707.84 for every additional year of experience, controlling for having a clinical doctorate (ß=0.454; p < .01). Experience adjusted for having a clinical doctorate as a model explained roughly 27% of the variance in salary (R^2^ = 0.265). Having the HEC-C was excluded from this model because it was not significantly predictive of pay when controlling for clinical doctorate and years of experience.^3^ However, compensated individuals have disproportionately high representation among those who have or plan to get the HEC-C in the next year (𝝌^2^ = 4.81; p < .05); compensated clinical ethicists are 2.83 times more likely than uncompensated clinical ethicists to have or plan to get the HEC-C.

#### Compensation by Employment Model

Differences in pay by employment model are shown in Table 4. Notably, differences in pay by degree type *within* the full-time ethicist employment model category were not significant. Other subgroups within employment model categories were not evaluated due to small subgroup sizes.

Additional analysis was performed on respondents working within less prevalent employment models. Of the 9.8% (13/122) of respondents who reported partial buyout of their full-time position (as shown in Table 4, 11 provided salary data), there was an average percentage of FTE buyout of 0.30 (SD = 0.2), with a range from 0.10 to 0.80. Among respondents being paid a retainer, 3 were paid monthly, 1 quarterly, and 2 annually. The average retainer adjusted to a monthly rate was $3,260 (SD=$4,934.13) with a range from $83-$11,666.66. Among those respondents working hourly, the average number of weekly hours of 7.3 (SD = 8.8, with median of 5.0)^4^ and a range from 1 to 25. None of the hourly consultants reported a limit on the number of hours they can bill. Finally, 1 respondent reported charging a flat fee per consult of $325.

Importantly, a significant proportion of the sample reported performing healthcare ethics consultation on an uncompensated basis (24.1%; 32/133). Those respondents averaged 3.9 hours a week (SD = 2.6) with a range of 1–12 hours. Another 6.8% (9/133) of participants reported that consultation is a form of service inherent in the job duties of another position they hold, but is not compensated per se.

In terms of the proportion of different degree types among uncompensated labor, those with a clinical doctorate were overrepresented with 56.5% (13/23) uncompensated, while among those with a non-clinical doctorate only 13.2% (7/53) were uncompensated (𝝌^2^ = 15.87; p < .001). Compared to all others, those with clinical doctorates were 3.6 times as likely to do uncompensated clinical ethics consultation.

#### Satisfaction and Other Forms of Support

Most respondents were satisfied with their compensation, with 65.1% being slightly to extremely satisfied, while 28.6% reported being slightly to extremely dissatisfied.

Additionally, survey respondents reported receiving various other forms of support for healthcare ethics consultation from their institutions. For example, 62.4% (83/133) reported some form of professional development or conference travel reimbursement, with a reported average of $2,844.92 (SD=$2,726.10) and a range from $400-$13,000. A slim majority of respondents were provided office space (55.6%, 74/133) and 58.6% (78/133) were provided a work computer, while 31.6% (42/133) were provided a phone or tablet, and 89.7% (35/39) of those who had, or were planning to take, the HEC-C reported they had received, or would receive, reimbursement for taking the HEC-C exam. Other forms of support reported in response to an open-ended question included research funds, secretarial support, mileage reimbursement, periodical subscriptions, a book budget, and health or life insurance typical of full-time employment.

While most respondents were satisfied to some degree with their compensation, some nonetheless used the open-ended prompt at the end of the survey to express frustrations about employment models, compensation, and lack of institutional support. Related to compensation, one respondent who performed clinical ethics consultation as a volunteer while otherwise working in an academic position remarked, “This model is so very broken. My community loses physician-ethicists all the time because the hospital won’t pay down their time.” In agreement, a physician clinical ethicist remarked, “We provide this service out of a sense of professional obligation, but lack of pay sends a message of undervaluing the resource by the hospital administration.” This accords with our finding that clinical ethicists with clinical doctorates are over-represented in the uncompensated category above.

Another respondent, whose primary job role was administrative as a director of bioethics, noted that by comparison, administrators within ethics services were underpaid relative to directors of other services: “Healthcare ethics consultants are not as well compensated as other directors with the same experience, although we typically have more responsibilities and greater educational attainments.” Perhaps this reflects a perception among hospital administration that ethicists are more clearly comparable to other support services in the hospital (e.g. social work), in ways that do not account for the disproportionately high level of educational attainment that many clinical ethicists represent; after all, the vast majority of our sample had doctoral level degrees of one form or another. Another physician-ethicist observed that the undervaluing of clinical ethics by administration had its origins at a broader, national level, writing, “The lack of national insurance-based or external financial incentives for hospitals to have a functioning ethics committee, such as pay for performance or professional fee billing, has a clear impact on executives’ interest in funding clinical ethics consultation.”

## Discussion

In sum, our data suggest that annualized healthcare ethics compensation averages around $150,000 per year, but that degree, professional role (insofar as several physicians worked on a buy-out model), and years of experience significantly affect compensation. The vast diversity of employment models, consultant education and experience, and other professional roles (particularly for buy-out models) make it challenging to estimate compensation rates for clinical ethics consultants. However, our data provide a starting point for identifying appropriate compensation, with the expectation that compensation levels could be adjusted based on degree and years of experience. We hope this information will be useful to both healthcare ethics consultants and healthcare institutions as they assess and negotiate fair compensation for this important work.

### Training, Employment Models, and Compensation

Our data build on the work of Danis et al. (2021), who report on the number of individuals receiving compensation for work in healthcare ethics programs, but do not identify compensation levels. Our data reveal tremendously wide variation in compensation, not only across the aggregated group, but also within subcategories of employment type and highest degree. Our study may prompt further discussion and research on compensation models that could inform greater standardization both of employment structure and pay rate in the field. An evidence-based picture of employment models and fair market compensation for clinical ethicists may also spur growth in the field as hospital administrators gain a clearer understanding of how an ethics service may be structured and what it will cost.

Despite wide variation in compensation, some trends were identifiable in our data. Clinical ethicists with a clinical doctorate as their highest degree most commonly provided ethics consultation on part-time/buy-out or volunteer bases. This indicates that physicians who provide clinical ethics services often do so as a part of, or a supplement to, their primary position as a clinician. Similarly, only 8 of the 30 respondents with clinical doctorates have any degree focused on clinical ethics. However, another 8 of those with clinical doctorates, but without bioethics focused degrees, had received, or were in the process of receiving, other kinds of advanced clinical ethics training. This suggests that those with clinical doctorates rely heavily on other kinds of clinical ethics education (e.g. short-term intensive courses).

Furthermore, the difference in pay across those with a non-clinical doctorate and a master’s as their highest degree suggests that some financial value is placed on possession of a terminal degree in clinical ethics. Formal training for clinical ethicists will become increasingly important as the field further specifies educational needs (Fox, Tarzanian, Danis, and Duke, 2021b) and develops best practices and standards, such as those articulated by the *Core Competencies for Healthcare Ethics Consultation* (American Society for Bioethics and Humanities, 2011). Further, our data suggest that greater effort should be invested in formal ethics education and training that can accommodate the needs of those primarily working in clinical positions, who tend to utilize non-degree types of clinical ethics training to a greater degree.

### Satisfaction and Support

Some respondents indicated dissatisfaction with their employment and compensation model and they identified a lack of support from leadership. This may be, in part, because the Joint Commission on Accreditation of Healthcare Organizations underspecifies the “framework” it requires for addressing situations of moral uncertainty or conflict. In particular, the Joint Commission’s requirement could be satisfied by an ethics committee of volunteers or an ethics service staffed by full-time, experienced, doctoral-trained ethicists. This may also be, in part, because of the lack of external financial incentives. In short, one obstacle in the professionalization of clinical ethics has been the need to build strong support from local leadership rather than a push from external financial or regulatory organizations. Support from local leadership has worked in some areas, but not others. If the discipline is going to professionalize on a national level, it may be time for professional organizations such as the American Society for Bioethics and Humanities or the Association of Bioethics Program Directors to advocate for support from external financial or regulatory bodies.

### Limitations

Because recipients of the survey invitation through CECAG (The Clinical Ethics Consultation Affinity Group) were encouraged to forward the link to other healthcare ethics consultants, it is difficult to estimate the response rate for this survey. The Clinical Ethics Consultation Affinity Group (CECAG) listserve has 967 members, which suggests that the response rate was low. However, not all CECAG members may be actively involved in healthcare ethics consultation, and some may have self-selected out of participation after reading the invitation. Nonetheless, our study may be underpowered to detect some group differences.

Relatedly, this study is limited by the complexity of its key variables. It is challenging to capture the diversity of practice contexts, employment models, and rates of compensation among such an interdisciplinary group, working in a diversity of employment models, who often do healthcare ethics consultation as an adjunct to one or more other roles, and in different proportions across distinct kinds of healthcare ethics consultation work (i.e. case consultation, education, and policy review). This presents challenges analytically, where joint frequencies complicate prevalence estimation and where analyses require partitioning the sample into mutually exclusive categories. Our choices about how to deal with these complexities have been described above, but the interpretation of the data should bear those in mind.

The sample is also not likely representative of healthcare ethics consultants nationally. Individuals subscribing to the CECAG listserve likely reflect a more academic subset of healthcare ethics consultants. As such, they are more likely to have doctoral degrees and formal training in clinical ethics, as well as more likely to hold academic appointments. Additionally, while Fox and colleagues have reported on ethicists working in for-profit hospitals (Fox et al., 2021a), the selectivity inherent in our sampling design may have yielded overrepresentation of clinical ethicists in non-profit or academic medical centers. In turn, our sample may overrepresent factors associated with academic medical centers such as being located in an urban area or how much time participants reported spending on educational activity (Danis et al., 2021). Future research estimating employment and compensation models for clinical ethicists in the for-profit sector is needed.

Finally, the lack of a standardized vocabulary for describing compensation models may have undermined the reliability of some of the results. For example, it is possible that some respondents who identified as “volunteers” are receiving *informal* release time from a full-time hospital position to facilitate their involvement with clinical ethics work (which would reflect a kind of compensation arrangement). For others, ethics consultation may simply be an inherent part of their job duties and therefore not compensated in particular, but be nonetheless associated with that respondent’s compensation package. However, only one respondent indicated they were both a volunteer and that ethics consultation was inherent in their job duties, noting that it was part of their committee service. This suggests there was not widespread misinterpretation of the meaning of doing “volunteer” healthcare ethics consultation.

## Conclusions

This paper provides data about compensation models for providers of clinical ethics services. However, it has also identified other information, such as trends in qualifications of volunteers and common settings of clinical ethics consultation. Our data are informative as it highlights variation in compensation models in the field.

Because the variability in pay is associated with intersecting factors such as degree level, degree type, and employment model, it is very challenging to provide an accurate estimate of compensation for clinical ethicists. Furthermore, there is presently an insufficient taxonomy and terminology to describe different employment models, as institutions devise diverse arrangements for healthcare ethics consultation. As the field professionalizes, we anticipate that the norms and standards for healthcare ethics consultation may converge on some employment models over others. Combined with greater utilization of trained healthcare ethics consultants, this may drive greater standardization of compensation rates, as seen in other professions, which often provide members more reliable compensation estimates.

Future efforts of both researchers and professional societies should focus on identifying and characterizing employment models, assessing their respective strengths and weaknesses, and offering recommendations for suitable healthcare ethics service structures, calibrated to the needs of different kinds of institutions and practice settings. This work would build on foundational research initiated by Gremmels (2020) and Repenshek (2021). While our paper provides pay estimates among many common employment model subtypes, conducting qualitative studies that allow participants to articulate their healthcare ethics employment experiences could contribute to a well-grounded taxonomy. By analyzing these narratives, we could establish a lexicon to standardize terminology, bolstering the scope and representation of future research.

## Footnotes

1 We recognize that this framing does not adhere to the proper distinction between sex and gender, however, other constructions of this question can complicate data accuracy because not all respondents understand the categories and distinctions in other formulations of this question. Our strategy therefore utilized a clear open-ended option for anyone who wanted to simply describe their gender identity.

2 In this instance, “highest” should not be read as a normative judgment about the value of each degree type. Instead, it refers to the degree that most likely anchors one’s employment and job functions.

3 One respondent reported receiving a 3% raise upon successful completion of the HEC-C program.

4 We report the median here because of extreme skewness in the distribution of this variable that cannot be corrected because of the small subgroup size.
