# Correction to: It’s Worth What You Can Sell It for: A Survey of Employment and Compensation Models for Clinical Ethicists

**DOI:** 10.1007/s10730-023-09513-2

**Published:** 2023-08-17

**Authors:** Jason Adam Wasserman, Abram Brummett, Mark Christopher Navin

**Affiliations:** 1 Department of Foundational Medical Studies, Oakland University William Beaumont School of Medicine, Rochester, MI USA; 2 Department of Pediatrics, Oakland University William Beaumont School of Medicine, Royal Oak, MI USA; 3 Clinical Ethics, Corewell Health William Beaumont University Hospital, Royal Oak, MI USA; 4 Department of Philosophy, Oakland University, Rochester, MI USA

The article “It’s Worth What You Can Sell It for: A Survey of Employment and Compensation Models for Clinical Ethicists“, written by Jason Adam Wasserman, Abram Brummett and Mark Christopher Navin, was originally published electronically on the publisher’s internet portal on 05 August 2023 without open access. With the author(s)’ decision to opt for Open Choice the copyright of the article changed on 08 August 2023 to © The Author(s) 2023 and the article is forthwith distributed under a Creative Commons Attribution 4.0 International License, which permits use, sharing, adaptation, distribution and reproduction in any medium or format, as long as you give appropriate credit to the original author(s) and the source, provide a link to the Creative Commons licence, and indicate if changes were made. The images or other third party material in this article are included in the article’s Creative Commons licence, unless indicated otherwise in a credit line to the material. If material is not included in the article’s Creative Commons licence and your intended use is not permitted by statutory regulation or exceeds the permitted use, you will need to obtain permission directly from the copyright holder. To view a copy of this licence, visit http://creativecommons.org/licenses/by/4.0/.

The original article has been corrected.

